# Illuminating Targets of Bacterial Secretion

**DOI:** 10.1371/journal.ppat.1004981

**Published:** 2015-08-06

**Authors:** Roger D. Pechous, William E. Goldman

**Affiliations:** Department of Microbiology and Immunology, University of North Carolina, Chapel Hill, Chapel Hill, North Carolina, United States of America; Geisel School of Medicine at Dartmouth, UNITED STATES

## Bacterial Secretion

The ability to secrete proteins is important to the pathogenesis of many bacteria. For gram-negative bacteria, the secretion system must deliver cargo through both an inner and outer membrane to reach a potential target. To date, there are six known gram-negative bacterial secretion systems, designated types I–VI secretion. For many highly pathogenic bacteria including *Yersinia pestis* and *Salmonella typhimurium*, secretion of protein effectors directly into target host cells is essential for virulence. Proteins secreted via the type III, IV, and VI pathways result in direct transfer of proteins across the host membrane and into the cytosol, and these systems will be the focus of the technology highlighted in this article.

Although the function of effector proteins secreted by these systems varies among different pathogens, common virulence mechanisms are evident. One common function of many secreted virulence factors is the targeting of host cytoskeletal function in order to promote uptake or inhibit phagocytosis. Another is modulating host cell cytotoxicity by inhibiting or promoting cell death in order to suppress innate immune function or to establish a replicative niche. Finally, an important mechanism common to many secreted effectors is the manipulation of host immune signaling. Until recently, fully evaluating the functional targets of bacterial secretion *in vivo* during infection was extremely difficult.

## FRET-Based β-lactamase Substrates as a Molecular Biology Tool

Förster (fluorescence) resonance energy transfer (FRET) has been used extensively as a cell biology tool to monitor the dynamics of intermolecular interactions within cells. FRET employs a donor fluorophore and an acceptor fluorophore in close proximity, and the emission spectra of the donor overlaps with the absorption spectrum of the acceptor. Excitation of the donor fluorophore results in resonance energy transfer to, and emission from, the acceptor. Disruption of the proximity between the two fluorophores results in strong emission from the donor upon excitation. The utility of FRET for measuring inter- and intramolecular interactions has been evident for some time. Using fluorescence as a measure of proximity, fluorophores exhibiting FRET can be incorporated to measure protein-folding dynamics in real time. Further, labeling separate molecules allows for measuring protein–protein interactions, the distance between molecules, and determining protein localization within a cell.

In 1998, Zlokarnik et al. used the gene encoding a common β-lactamase along with a FRET-based substrate to isolate individual cells with defined transcriptional responses from within a population of mammalian cells [1]. Zlokarnik et al. designed and synthesized the membrane-permeant ester CCF2/AM, which consists of a 7-hydroxycoumarin donor fluorophore and a fluorescein acceptor linked by a cephalosporin antibiotic. In the intact molecule, donor excitation at 405 nm (violet light) will result in fluorescein acceptor emission as green light at 520 nm that can be detected using flow cytometry or fluorescence microscopy. In the presence of β-lactamase, the cephalosporin linker is cleaved and the 7-hydroxycoumarin moiety is free from FRET, resulting in emission at 447 nm (blue light). Importantly, CCF2/AM is nonpolar and readily crosses mammalian cell membranes. Once in the cytosol of mammalian cells, the CCF2/AM ester group is hydrolyzed, trapping CCF2 and preventing leakage out of the cell. Thus, activation of a promoter of interest cloned upstream of the β-lactamase gene can be identified by incubating a cell population with CCF2/AM and screening for blue fluorescence.

## β-lactamase Hybrids to Monitor Bacterial Secretion In Vivo

**Fig 1:**
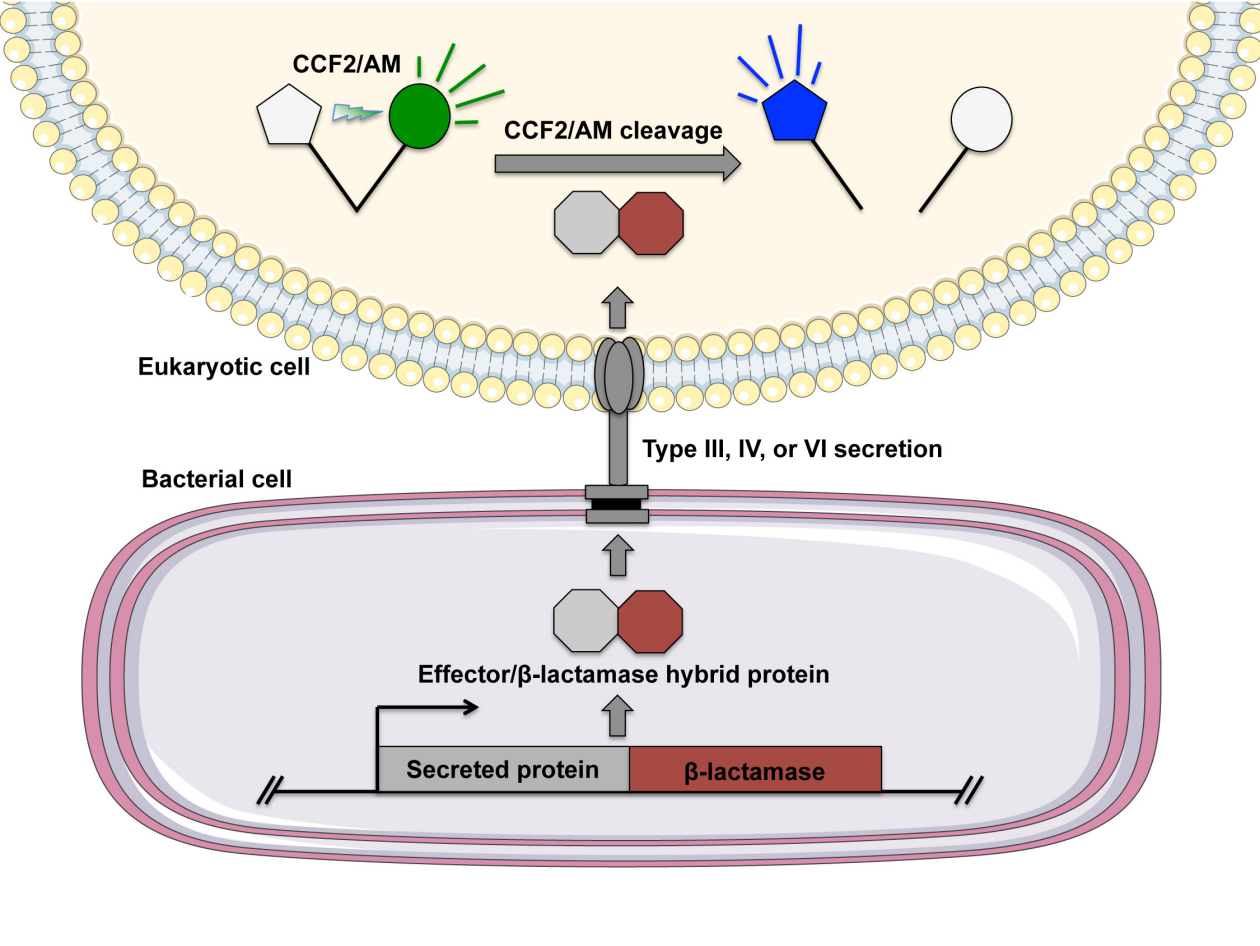
Evaluating bacterial secretion using the FRET-based substrate CCF2/AM. To monitor bacterial secretion, the gene encoding a β-lactamase is cloned in-frame with a portion of the gene encoding a predicted secreted protein to generate a translational fusion. Eukaryotic cells are infected with the bacteria harboring the fusion protein, followed by incubation of infected cells with the fluorescent substrate CCF2/AM (or its analog CCF4/AM). CCF2/AM consists of 7-hydroxycoumarin and fluorescein connected with a cephalosporin linker. Cells can then be examined by flow cytometry, fluorescence microscopy, or using a plate reader in a multiwell format. Under excitation at 405 nm, the 7-hydroxycoumarin and fluorescein moieties of CCF2/AM exhibit FRET, and emission is detected from the acceptor fluorescein as green fluorescence (520 nm). If the target cell has undergone translocation and harbors the bacterial fusion protein, the cephalosporin linker is cleaved by β-lactamase, which disrupts FRET and results in blue fluorescence from 7-hydroxycoumarin at 447 nm.

In 2004, Charpentier and Oswald demonstrated the utility of β-lactamase–protein hybrids for analyzing bacterial secretion [2]. Briefly, a sequence encoding the common β-lactamase TEM-1 was fused to the C-terminus of known secreted effectors in enteropathogenic *Escherichia coli*, and the resulting strains were used to infect HeLa cells. HeLa cells were then incubated with CCF2/AM, and translocation of effectors was visualized using fluorescence microscopy, in which cells that had undergone effector translocation exhibited blue fluorescence (Fig 1). In 2005, Marketon et al. extended this technique to identify cells targeted by *Y*. *pestis* type III secretion in vivo [3]. Until this point, identifying and measuring the host cells targeted for secretion during infection had been nearly impossible, requiring identification of a relatively small cell population within an entire animal. The authors infected mice with *Y*. *pestis* strains carrying translational fusions between secreted effector *Yersinia* outer proteins (Yops) and the functional region of TEM-1 β-lactamase, and they were able to detect Yop translocation as blue fluorescence during infection. To do this, Marketon et al. harvested the spleens of infected mice and subjected splenocytes to flow cytometry using a panel of fluorescently labeled antibodies to differentiate between T cells, granulocytes and neutrophils, dendritic cells, and macrophages. Using this method, Marketon et al. were able to measure and quantitate Yop translocation in vivo during infection, resulting in the discovery that *Y*. *pestis* primarily targets cells of the innate immune system for Yop translocation. Since this initial work, this approach has been used to examine the secretion of bacterial effector proteins for a number of bacteria including enteropathogenic *Yersinia* [4,5], *Salmonella* [6], *Pseudomonas* [7], and *Legionella* [8] species. Thus, the ability to monitor bacterial secretion in vivo during infection has allowed for direct analysis of host–pathogen interactions during infection and has allowed for an unprecedented look at how pathogenic bacteria target the host to establish and maintain infection.

## Extending Utility beyond the Identification of Target Cell Types for a More Detailed Evaluation of Secretion

Since the initial in vivo studies, β-lactamase–protein hybrids coupled with FRET substrates have allowed for an increasingly detailed examination of bacterial secretion. Detection techniques have been refined to enable precise quantitation of secretion on a per-cell basis, allowing for the identification of mutants affecting the rate and fidelity of effector secretion [9]. In addition, the lactam ring of CCF2/AM has been modified to create CCF4/AM, which has improved solubility and slightly better FRET and is therefore better suited for screening applications. The fluorescent readout of translocation coupled with mutagenesis of bacterial genes has allowed for the detailed evaluation of translocation kinetics and mechanisms for a number of pathogens, as well as the identification of genes that contribute to effector secretion [10–12]. Of note, the robust fluorescent signal associated with the commonly used FRET substrates lends itself to the design of high throughput assays for the identification of mutants or factors that alter translocation in tissue culture. One potentially important application of this has involved the implementation of assays to identify inhibitors of bacterial secretion for the development of potential therapeutics [13,14]. As the secretion of effectors is essential to the virulence of a number of highly pathogenic gram-negative bacteria, the bacterial secretion machinery and/or secreted effectors may prove effective as new therapeutic targets [15].

## Using β-lactamase/FRET Substrates to Probe Host Elements of Pathogenesis

An important application of FRET-substrate–effector β-lactamase hybrids has been for identifying host factors that contribute to effector translocation. This has primarily been accomplished by examining effector translocation in the presence of inhibitors of host function or coupled with the silencing of host genes [16,17]. For example, Newton et al. utilized small interfering RNA (siRNA) silencing to examine the role of specific host Rab GTPases in type IV secretion of effectors for the bacterium *Coxiella burnetii* [16]. Similarly, Sheahan et al. combined FRET of a Rho GTPase biosensor with flow cytometry in an RNA interference (RNAi) screen to identify host factors involved in type III secretion of *Yersinia pseudotuberculosis*, revealing a role for the chemokine receptor CCR5 in translocon function [18]. Coupling detection of translocation with methods to deplete specific host cell populations has contributed to the understanding of mechanisms that govern target cell preference in vivo during infection [5,19]. This approach was perhaps most elegantly employed by Durand et al., who used neutrophil depletion studies to show that the presence of professional phagocytes dictated the number of host cells targeted for Yop translocation by the enteric pathogen *Y*. *pseudotuberculosis* [5].

A major hurdle to studying the effects of bacterial secretion in vivo is the difficulty of efficiently isolating target cells from infected animals for subsequent analysis. One innovative approach addressed this issue by utilizing the β-lactamase/FRET system to identify and isolate host cells for further study. Using flow cytometry to sort cells from the spleens of mice infected with *Y*. *pseudotuberculosis*, Rolán et al. identified cells that had been injected with the effector protein YopH, a potent tyrosine phosphatase, and separated these from cells that had not been injected [20]. These two-cell populations were collected and subsequently analyzed by western blot to identify specific targets with decreased phosphorylation in the presence of YopH. Expanding this analysis to evaluate host responses to effector intoxication in greater detail will likely provide crucial information regarding the direct host–pathogen interactions that occur during infection. Thus, the use of bacterial effector–β-lactamase hybrids along with FRET-based substrates has proven ideal for evaluating host–microbe interactions and has allowed for detailed evaluation of both the bacterial and host factors that contribute to a process essential to virulence of many pathogenic bacteria. In the future, this technique will continue to be highly useful in high-throughput approaches for studying both microbial and host cellular processes that contribute to infection, particularly for the identification of inhibitors that might have therapeutic applications. Further, the coupling of FRET-based substrates with fluorescence-activated cell sorting has great potential for the isolation and analysis of specific target cells from infected organs, providing a high-resolution view of host–pathogen interactions during infection.
